# Problem-based learning in resource-poor settings: lessons from a medical school in Ghana

**DOI:** 10.1186/s12909-015-0501-4

**Published:** 2015-12-14

**Authors:** Daniel Amoako-Sakyi, Harold Amonoo-Kuofi

**Affiliations:** 1 Problem-Based Learning (PBL) Unit, School of Medical Sciences, College of Health and Allied Sciences, University of Cape Coast, Cape Coast, Ghana; 2 Provost, College of Health and Allied Sciences, University of Cape Coast, Cape Coast, Ghana

**Keywords:** PBL implementation, Medical education, Resource-poor settings, Ghana, UCCSMS

## Abstract

Problem-based learning (PBL) is arguably one of the most important innovations in medical education in the last century. The evident benefits of PBL and the changing face of medicine and medical education have led many institutions including those in resource-poor settings to consider the adoption of PBL curricula. However, experts are uncertain about how successful PBL will be in such settings, as literature on the implementation of PBL in resource-poor settings appears to be inadequate. The University of Cape Coast is located in a resource-poor setting, however, its medical school has used PBL curriculum since 2007. In a descriptive prose, this article discusses the PBL implementation processes, the challenges faced, the mitigation strategies employed, and the lessons learned at University of Cape Coast School of Medical Sciences (UCCSMS). The arguments fall under the broad themes of curricular structure, resource constraints, faculty development, and assessment. The peculiar socioeconomic situation of Ghana, challenges in funding of tertiary education, and the resource implications of PBL provided the context for the arguments. It emerged out of the discussion that PBL has to be implemented as whole curriculum to be effective. Regular faculty development activities on PBL and the alignment of assessment methods with PBL also emerged as important issues in the discussion. The article argues that in spite of its cost implication, a PBL curriculum can be successfully implemented in resource-constrained settings.

## Background

Problem-based learning is not a new concept but over the last few decades, it appears to have become the preferred pedagogical strategy in tertiary education worldwide. Many universities and faculties have shifted to PBL in the bid to make their programs student-centred, multidisciplinary, and professionally relevant [1, 2]. Evidence suggesting that PBL has several advantages over the traditional system is the driving force for this shift in most instance. Apart from encouraging students to actively participate in their own education, PBL enhances critical thinking and promotes collaboration among students [3]. Problem-based learning inherently allows the integration and organization of learned information for easy recall and application. This makes PBL graduates lifelong learners with the cognitive flexibility to use varied knowledge sources in solving different problems [2, 4].

Championed by McMaster University in Rochester, Canada in the 1960s, PBL has since spread widely in the last five decades to reach every continent. From McMaster, through early adopters like University of Maastricht, in the Netherlands; Newcastle University, in Australia and University of New Mexico, in the USA; to late comers like University of Cape Coast School of Medical Sciences (UCCSMS) and University for Development Studies School of Medicine and Health Sciences in Ghana, PBL has evolved into many variants. The emergence of several variants of PBL is partly due to the peculiar implementation constraints and opportunities that exist in different universities [2]. Variants of PBL curricula has been classified into four main types [5]. In this classification, type I PBL curriculum is primarily cosmetic with a few PBL problems/scenarios per academic year. This type uses PBL scenarios as ornaments under a conventional curriculum driven by teaching via didactic lectures. The type IV PBL curriculum, on the other hand, is one that uses PBL as the main learning platform and judiciously employs interactive lectures and other didactic sessions to enrich concepts and further motivate students for self-directed learning. The type IV PBL curriculum, although a hybrid, is referred to as a standard PBL curriculum by some authors whiles others opine that a “pure” form of PBL has now gone extinct [2, 5]. Between the two ends of the spectrum are types II and III PBL curricula that are deemed better than the type I but falls short of the ideal type IV PBL curriculum.

The School of Medical Sciences in the University of Cape Coast (UCCSMS) adopted a PBL curriculum in 2007 for its MB ChB program. The School has since graduated two batches of medical doctors. It is difficult to conduct an objective comparative study of medical school graduates from different universities in Ghana since there is no common qualifying examination for medical school graduates. However, anecdotal evidence suggests that the medical education community in Ghana have a positive perception of the PBL curriculum used by UCCSMS. In addition, the performance of UCCSMS students in national health-related extracurricular activities suggests that their knowledge and communications skills compares favourably with their counterparts [6]

In spite of the many advantages of a PBL curriculum, resource implications sometimes pose a major challenge to its implementation and maintenance. The provision of specially equipped tutorial rooms, recruitment of adequate numbers of qualified faculty, periodic faculty development activities, and a myriad of logistic requirements threatens to put PBL out of the reach of resource-constrained universities, particularly those in middle and low-income countries where the budget for education is often inadequate. This stokes the debate on whether a functional PBL curriculum can be implemented effectively in resource-poor settings. [7, 8].

Literature on the implementation and use of PBL in Sub Saharan Africa is limited and disproportionately represents institutions from South Africa, which is neither a low nor a middle-income country [9–13]. The University for Development Studies (UDS) in Ghana uses PBL but it is unclear from the literature which type of PBL curriculum was implemented and the implementation challenges they have faced. This article gives a concise descriptive account of the implementation of a PBL curriculum at UCCSMS. It has three main parts: a description of the UCCSMS curricular structure, the implementation challenges faced, and how the implementation challenges were mitigated. This article provides valuable insight and guidance to institutions planning to implement PBL and those thinking about improving dysfunctional PBL curricula.

## The PBL curricular structure; more than pedagogy

Problem-based learning is more than pedagogy – it is a curriculum [14]. Thus, the implementation of PBL goes beyond the mere addition of “PBL-like” activities to a traditional curriculum. A PBL curriculum may be implemented in one of two ways: 1) either an existing conventional curriculum is transformed into PBL curriculum or 2) a PBL curriculum is implemented from scratch with a new programme. Both pathways present formidable challenges, however, starting from scratch presents additional PBL unrelated challenges. UCCSMS implemented a PBL curriculum from scratch – but regardless of the pathway, key issues pertaining to infrastructure, human resource, logistic, and the PBL curriculum to be implemented needed to be thoroughly considered.

### The UCCSMS curricular structure

The curriculum at UCCSMS does not follow the traditional system. It adopts an educational philosophy that makes integration of content across disciplines and the elimination of the so-called basic/clinical sciences divide a central theme. The structure follows a student-centred, problem-based, and integrated format with strong emphasis on community orientation. The main areas of the curriculum are covered using a body-system approach, which allows the programme to be organized into system-based modules in a spiral format. It is a hybrid curriculum comprising a careful blend of PBL activities and didactic sessions. The curriculum is rolled out in a 5-year programme that is preceded by a foundation or premedical year (level 100). The aim of the foundation year is to bridge the gap between Senior High School (SHS) and University. It also prepares students for academic work in the medical school with modules carefully designed to help students develop the essential intellectual and social skills required to study medicine. Faculty who teach level 100 courses are drawn from several departments in the university and the mode of delivery does not include PBL. Levels 200 and 300 make up the first cycle of the curriculum, which is designed to foster an integrated understanding of basic human structure and function in health and disease. The PBL scenarios used at this level are designed to help students learn basic science concepts whiles clinical concepts are reinforced by weekly sessions at the clinical skills laboratory. A PBL cycle (time taken to complete a scenario) in cycle I is one week with two tutorial group meetings and a plenary presentation. Typically, the first and second tutorial meetings are held on Mondays and Wednesdays respectively while plenary presentations by the various PBL groups are held on Fridays. The number of PBL scenarios per module depends on the length of the module. Levels 400, 500 and 600 make up the curriculum cycle II, which consists of a mixture of body-systems and other modules. In cycle II, emphasis is placed on clinical intervention and management with the basic science content reduced to the essential minimum. Students are divided into small groups and attached to clinical specialities in rotation during cycle II. PBL scenarios in the cycle II help students to learn the various clinical interventions and the science that underpins them. The PBL cycle for cycle II is one and a half weeks and tutorial group meetings are held on Mondays and Fridays. Level 600 students do not have plenary presentations.

**Fig. 1:**
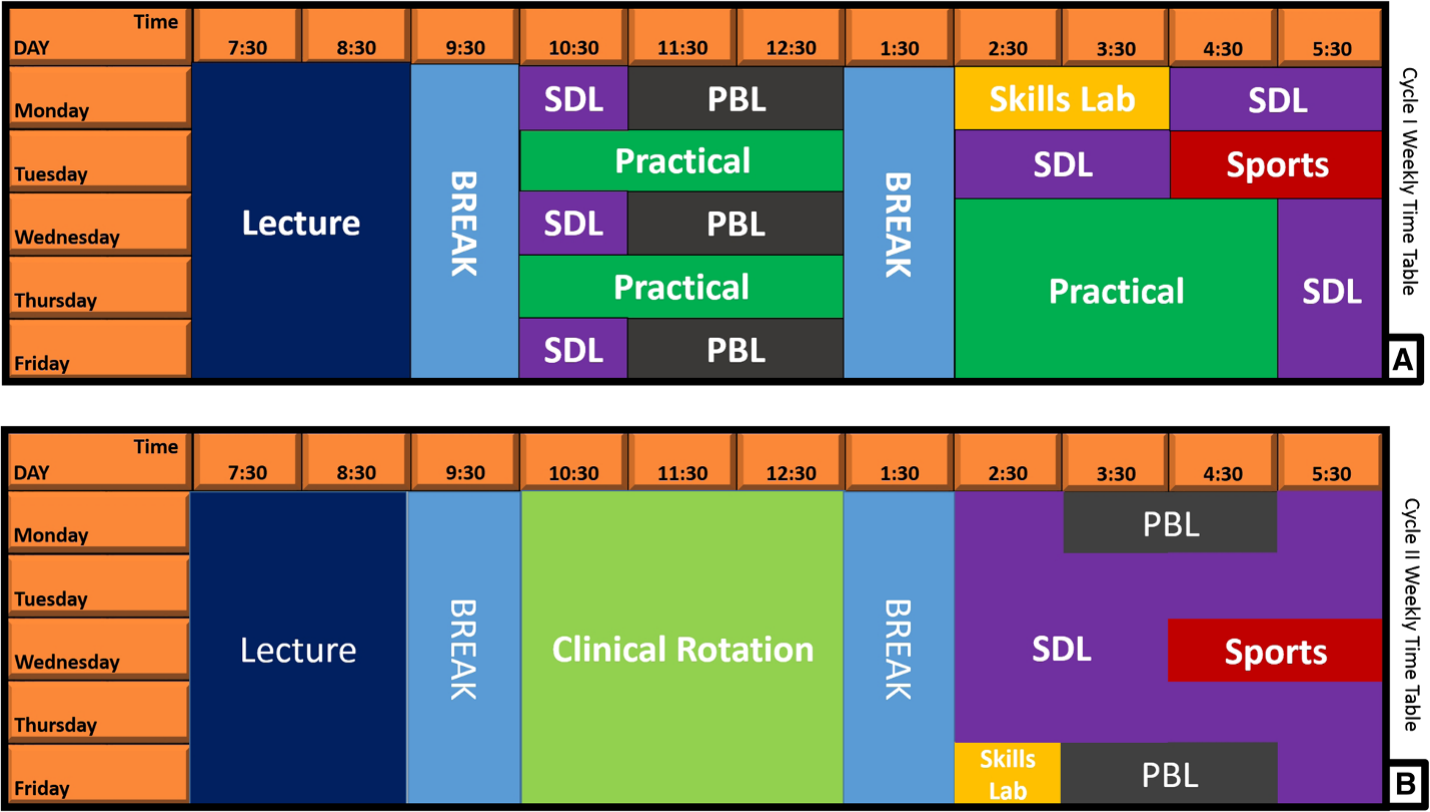
Weekly time table templates for cycles I and II. A weekly time table template showing the various teaching and learning activities and their time allocations. Time for activities that are not applicable in specific modules coverts to SDL

Teaching and learning methodologies used in this curriculum include PBL tutorials and seminars; practical sessions including anatomy dissections, laboratory work and simulations; structured clinical skills training; lectures; and community field work. These methods are carefully deployed in a complementary manner for optimal impact. Lectures are usually held in the first two hours of a school day and they usually constitute 20% of the available teaching time per week. The remaining 80% of available teaching time are used for non-lecture-based teaching and learning activities including student-directed learning (SDL) (Fig. 1). Community-Based Experience and Service (COBES) and Clinical Skills Training are two other important features of this curriculum. The objectives of the COBES program is to provide broad-based people-centred education that train doctors to have a strong community orientation. Levels 200 to 500 students go on COBES for a four-week period each second semester at approved COBES sites. During those 4-week period, students live on site (in the communities) and devote their time to work in the community. Clinical skills training takes place at the Maersk Clinical Skills Laboratory which is a safe, friendly, and controlled learning environment where students practice and perfect clinical skills, communication and technical procedures.

## Implementation challenges

### Curriculum design

An essential first step in implementing a PBL curriculum is to have a curricular document in place. In line with the PBL philosophy, the implementation committee at UCCSMS set out to design a curriculum that allows active student participation and places content to be learned in a relevant clinical context. To achieve this, we adopted the Maastricht “seven-jump” PBL format [15] and modified it slightly to incorporate a plenary seminar session. This plenary seminar brings together the various tutorial groups to present the content learned in that PBL cycle to colleagues and faculty. The rationale behind this plenary session is to equip students with presentation skills and afford faculty the opportunity to correct misconceptions. In a hybrid PBL curriculum, it is essential for the curriculum document to clearly outline and delimit the roles of other teaching and learning activities to avoid unnecessary overlaps. Finally, it is imperative to institute a proper curriculum governance to ensure that curriculum is being implemented properly. At UCCSMS, the Curriculum committee works together with other committees to ensure that the objectives of the curriculum are adhered to.

The development of the UCCSMS curriculum involved a number of retreats with content experts. The contribution of various clinical and basic scientists and that of medical educators to the curriculum cannot be overemphasized. These, notwithstanding, most of these experts were unfamiliar with PBL and therefore opposed some of the ideas, not because the ideas lacked substance, but rather because the experts could not fully appreciate those ideas. This situation has to be managed well to avert the potential of ending up with a dysfunctional curriculum with a PBL façade. It might be necessary to precede such curricula design retreats with a PBL workshop to introduce and tune the minds of content experts to the objectives of the retreat. Getting the PBL curricular document ready is an important first step; however, the implementation of the curricular document is daunting and requires the cooperation of faculty members, students, non-academic staff, and the wider university community.

### Resource limitations

In a PBL curriculum, students study in small groups of about 6 – 10 members and each of these groups is assigned a faculty member (facilitator) whose role is to facilitate the learning process at the tutorial group meetings. The human resource implication of this setting is massive: - a class of 40 students will need at least four facilitators (lecturers) instead of one. To put things in perspective, UCCSMS currently uses PBL at five levels (from 200 to 600) with each level having 4 to 6 tutorial groups. This translates into 25 groups requiring 25 facilitators (lecturers) during tutorial group meetings. For larger classes of 100 or more, the requirement for facilitators can be daunting. This constitutes a huge increase in the cost of running a PBL curriculum as compared to lecture-based learning (LBL). The debate as to whether this excessive faculty cost is justified is ongoing. However, a study to evaluate this cost-benefit question concludes that PBL does not place unreasonable demands on the time of faculty [16].

The other resource implication of a PBL curriculum is infrastructure. Directly related to having tutorial groups is the need for tutorial rooms. Ideally, PBL tutorial rooms should be purpose-built and well designed with adequate illumination and ventilation to provide a pleasurable learning environment for students and faculty. Poorly lit and ventilated tutorial rooms could make student uncomfortable and eager to end tutorial sessions before time [17]. Here again, more rooms are needed for a PBL curriculum as opposed to an LBL one. For instance, at UCCSMS, we had to come up with enough tutorial rooms to accommodate all of our 25 tutorial groups. This is a challenging task even when tutorials are scheduled at different times to allow multiple tutorial groups to use a single tutorial room. Adequate learning resources including well-resourced libraries, reliable internet connectivity, functional clinical skills laboratories, and basic science laboratories facilitate self-directed learning (SDF) in a PBL curriculum. In spite of being vital to a PBL curriculum, the provision of these facilities is an uphill task in resource-poor settings. Similarly, the provision of logistics such as flip charts, markers, projectors, etc. is an additional strain on the inadequate education budget in such settings.

### Dealing with tutors

Becoming a PBL tutor represents a significant change in role for some faculty members [3]. Some consider it a demotion from their acquired or ambitioned status as eminent academics to an insignificant facilitator of learning. For others, there are genuine feelings of uncertainty about their roles as PBL facilitators: they are unsure about how, how often, and when to intervene, how detailed and directive they should be, and whether they should be content experts or not. Faculty perceptions on PBL curricula are generally positive [18–20] but that notwithstanding, these issues need to be addressed to prevent faculty from becoming frustrated and opposed to the curriculum. A PBL training workshop for newly engaged faculty and periodic refresher courses for old faculty are crucial in this respect. At UCCSMS, signs of faculty’s frustration and opposition to PBL were implicit rather than explicit. It manifests occasionally as apathy and absenteeism from PBL tutorials. Faculty members who are unsure about PBL may also push to add more lecture slots to the weekly time table and in so doing rob students of their self-directed learning (SDL) time. Some may even attempt to schedule lectures at SDL periods without recourse to the module coordinator. Subsequent sections of this article will describe the faculty development strategy (with regards to PBL) at UCCSMS and how this threat has been dealt with.

### PBL scenarios

Scenarios also referred to as cases or problems, play a pivotal role in PBL. They are essentially learning objectives transformed into scenarios and they allow students to learn clinical, basic and behavioural science concepts in an integrated manner [21]. Thus, the success of PBL, as a learning or an educational strategy hinges heavily on the quality of the scenarios and the range of the scenario bank [22, 23]. Good scenarios integrate several disciplines, encourage discussion of cognitive domains, promote collaboration and encourage self-directed learning [22]. The most effective scenarios: (a) address one or more learning objective(s) in the module guide; (b) activates prior knowledge and builds on existing knowledge; (c) are commensurate with the level of the students; (d) are relevant to the future profession of students; (e) stimulate critical thinking and encourage self-directed learning.

Undoubtedly, getting PBL scenarios is not an easy task. It requires teams of educational and subject experts, staff training, and expensive writing retreats. Some universities avoid this stress and purchase PBL scenarios from elsewhere but purchased scenarios may be socially and culturally inappropriate and often require adaptation and further fine-tuning before they can be used. The number of scenarios needed per module/block will depend on the length of the block and the variant of PBL being used. UCCSMS generates its own scenarios and faculty is tasked with writing PBL scenarios for the various modules. Presently, only a few faculty members have written majority of the scenarios used in UCCSMS and although the PBL training for new faculty includes a session on scenario writing, majority of faculty are yet to write their first PBL scenarios. The factors that motivate faculty to write scenarios need further study.

### Assessment drives learning

The cliché: “assessment drives learning” has proven to be true in many settings, particularly in medical education [24]. However, institutions that use PBL curriculum appear not have adequately aligned their assessment with the educational philosophy of PBL. This undermines the effectiveness of PBL and dampens students interest in PBL since their efforts seems not to be rewarded. Assessment at UCCSMS is structured to match our curriculum and it spans three main categories: formative, continuous, and annual assessment [25]. By definition, formative assessments are non-scoring, however they provide valuable feedback for faculty and students and thus, they are obligatory. Continuous assessment constitutes 60% of student’s marks for the academic year and student’s assessment during PBL tutorial group sessions and presentation constitute 15% of the continuous assessment marks. This may still not be enough motivation if as sometimes happens, the majority of the questions require recall of facts rather than critical thinking and application of knowledge.

## Overcoming problems

The earlier section of this article alludes to the fact that implementation of PBL at UCCSMS faced some challenges. This section discusses how UCCSMS overcame some of the implementation challenges it faced. The authors also suggest ways to deal with chronic and emerging challenges associated with PBL.

### Laying a good foundation

Understanding from the onset that PBL is a curriculum and not just another teaching strategy is vital for its successful implementation. Envisioning PBL as a curriculum does not necessarily mean implementing a “pure” PBL – it rather refers to thoughtfully using interactive lectures and didactic sessions to scaffold student-centred, self-directed, and active learning which are triggered by carefully crafted scenarios/problems [2]. It might even be necessary to “hybridize” PBL to suit the economic and socio-cultural environment of the implementing institution [7]. “Hybridize” in this context is used advisedly to mean a Type IV PBL [5]. After a careful evaluation of our institutional environment and review of literature, the implementation committee of UCCSMS decided to adopt a uniquely hybridized PBL curriculum with Community-Based Experience and Service (COBES) as a key feature [26]. Making a decision as to what type of PBL curriculum to be implemented is an important first step. UCCSMS started as a new medical school - and this brings along some unique challenges that needed to be well managed. For instance, experienced professors who join new medical schools are often tempted to import bits and pieces of what they “considered” good practices in medical education. Although this is often done in good faith, it can potentially derail the PBL implementation process. The implementation committee at UCCSMS made the blue print of the curriculum to be implemented and the roadmap for implementation available to all newly appointed faculty. They were also required to attend a compulsory PBL training workshop regardless of their rank or experience with PBL. The latter, in particular, helped clarify misconceptions about the curriculum. From hindsight, the importance of introductory PBL workshops for participants of curriculum design/review workshops become patent.

### Overcoming the resource limitations

Financing tertiary education in most Sub Saharan African countries including Ghana is a big challenge for governments and stakeholders [27, 28]. This challenge is further aggravated by the concurrent increase in enrolment and the reduction in financial support from governments and international donors [29]. The constant underfunding of tertiary institutions in Ghana has led to a huge financing gap that has implications beyond the scope of this article. Attempts to reduce the financing gap led to the implementation of a cost-sharing mechanism in 1998 [30]. Cost-sharing in Ghanaian tertiary institution is still evolving and currently students in public universities make varied contributions to the cost of their education depending on whether they are fee-paying or non-fee paying [30]. Students of UCCSMS are all fee-paying but the tuition fee charged is only about 50% of the actual cost of training a medical doctor.

In Ghana, tertiary institutions are primarily funded through budgetary allocation received from the government through the National Council for Tertiary Education (NCTE) [30]. The mechanisms used in distributing funds to the various tertiary institutions includes: historical funding or incrementalism; bidding and bargaining; and discretion [30]. The last two mechanisms affords institutions the opportunity to lobby for additional funds over and above their allocations by making special cases in favour of a new programme, peculiar infrastructural needs, etc. The founding dean of UCCSMS and his team made use of this opportunity to substantially increase the funds allocated to UCCSMS. The team also managed to attract funds from private individuals and organisations for specific projects such as the Maersk Clinical Skills Laboratory and the Clinical Teaching Centre, all of which are vital for PBL. Although this does not address the larger underfunding problem in Ghanaian tertiary institutions, the ability to lobby government and private individuals and organisations for extra funds significantly contributed to the successful implementation of a PBL curriculum at UCCSMS. In many respects, putting in place a strong lobby, establishing linkages with industries, and having an efficient grantsmanship outfit appears to be vital for tertiary institutions regardless of the curriculum they run.

The provision of well-equipped PBL tutorial rooms is vital to the implementation of a PBL curriculum. At UCCSMS we did not have and still do not have purpose-built PBL tutorial rooms. Various spaces in the Clinical Skills Lab, Anatomy department, Cape Coast Teaching Hospital, and Diagnostic Centre have been converted to tutorial rooms. This has compromised comfort in some instances and hindered the incorporation of Information Communication Technology (ICT) into PBL scenarios, however, waiting for purpose-built tutorial rooms can unduly delay the implementation of a PBL curriculum. Our experience at UCCSMS suggests that safe, well-lit and well-ventilated rooms that are capable of comfortably seating up to 10 students may suffice as tutorial rooms. Another way of dealing with this problem is to schedule PBL tutorials for different levels at different times. This allows tutorial rooms to be used by multiple groups. It can however result in holding tutorial group meetings at odds hours to the discomfort of students and faculty. These interventions should be viewed as temporary and a long term plan of acquiring purpose-built PBL tutorial rooms pursued.

### Faculty development

The main problems of faculty in a PBL curriculum pertains to their own identity crises and the way they assess students. Shifting from a teacher-centred to a student-centred curriculum often represent change in identity that takes time to adjust to. Our half-day training workshop for newly appointed faculty is designed to introduce faculty to PBL and their new roles as facilitators of learning. This training workshop largely allays some of the apprehensions of new faculty but some become comfortable only after facilitating a few tutorial group meetings. Other faculty development activities undertaken at UCCSMS include workshops on scenario writing and how to write questions. Eight UCCSMS faculty members have so far attended PBL-related courses in Maastricht University and visited their facilities to appreciate at first-hand how PBL has been implemented elsewhere. These faculty members often form the core of facilitators used for our various training workshops.

In spite of these training workshops, the problems pertaining to assessment has persisted and it is one of the most difficult problem to deal with. Examinations for summative and annual assessment do not have the desired balance of integrated and application questions. This mismatch between learning and assessment undermines PBL and could be a source of discouragement for students. Workshops on writing test questions has improved the quality of questions but that notwithstanding, the desired balance of comprehension, applications of knowledge, analytical or critical thinking is yet to be achieved. This points to the need for training of faculty members on regular basis. Moderation of submitted questions to ensure quality and appropriateness is an effective way of matching learning with assessment. At UCCSMS, we have partly mitigated this problem by incorporating Objective Structured Practical Examination (OSPE) and Objective Structured Clinical Examinations (OSCE) into our end of semester and end of year examinations. OSPE/OSCE have several advantages over other modes of examinations. First and foremost it enables examiners to test the student’s ability to integrate knowledge, clinical skills and communication skills [31]. Furthermore, the flexible structure of OSPE/OSCE examinations make them easily adaptable to local needs [31, 32] .

### PBL scenarios

Our experience at UCCSMS so far suggests that the best way of getting new scenarios is to hold periodic scenario writing retreats for faculty. This is, however, very expensive and often cost prohibitive. Perhaps a viable alternative is for authorities to find innovative ways of motivating faculty to write scenarios such as making it count towards career progression.

## Summary

In spite of the ambivalence in literature, problem based-learning as a curricular innovation seems to have substantial and practical advantages over the conventional/traditional system of education [3, 33–35]. However, literature on the appropriateness of PBL in different cultural and socioeconomic settings is scarce. This article has provided insights as to how a PBL curriculum may be implemented in a resource-constrained setting. A descriptive account of the implementation challenges faced by UCCSMS has been given with a description of how some of these challenges were mitigated. Our experience and that of other institutions in similar settings suggests that the type of PBL curriculum is fundamental to a successful implementation. Institutions planning to implement PBL should avoid the pitfall of a cosmetic PBL i.e. a curriculum that is essentially traditional with elements of PBL only as an auxiliary pedagogic tool. That notwithstanding, an insistence on implementing “pure” PBL without recourse to local situations could present many formidable obstacles [7]. A hybrid or standard PBL curriculum that thoughtfully uses didactic sessions in a non-redundant manner may be a more feasible approach in these settings. Institutional leaders will have to constantly monitor and find pragmatic solutions, which may differ from one institution to the other, to mitigate the inexorable problems associated with the implementation of PBL.

## Abbreviations

ICT: Communication Technology
NCTE: National Council for Tertiary Education
OSCE: Objective Structure Clinical Examinations
OSPE: Objective Structure Practical Examinations
PBL: Problem-based learning
SDL: Student-directed learning
UCCSMS: University of Cape Coast, School of Medical Sciences
